# Supplementary material for: Transient Receptor Potential Canonical 5 (TRPC5): Regulation of Heart Rate and Protection against Pathological Cardiac Hypertrophy
Source: Biomolecules. 2024 Apr 4;14(4):442. doi: 10.3390/biom14040442 (PMC11047837; doi:10.3390/biom14040442)
Supplement: Supplementary file 1 [file biomolecules-14-00442-s001.zip › biomolecules-2914565-supplementary.pdf]

Article

Transient Receptor Potential Canonical 5 (TRPC5): Regulation of Heart Rate and Protection against Pathological Cardiac Hypertrophy

Pratish Thakore [1,2], James E. Clark [1], Aisah A. Aubdool [1], Dibesh Thapa [1], Anna Starr [1], Paul A. Fraser [1], Keith Farrell-Dillon [1], Elizabeth S. Fernandes [3], Ian McFadzean [2,4,*] and Susan D. Brain [1,*]

1. BHF Cardiovascular Centre of Research Excellence, School of Cardiovascular and Metabolic Medicine & Sci-ences, King's College London, London SE1 9NH, UK; thakore.pratish@gmail.com (P.T.); james.2.clark@kcl.ac.uk (J.E.C.); a.aubdool@qmul.ac.uk (A.A.A.); dibesh.thapa@kcl.ac.uk (D.T.); an-na.starr@gmail.com (A.S.); paul.fraser@kcl.ac.uk (P.A.F.); keith.farrell-dillon@kcl.ac.uk (K.F.-D.)
2. School of Cancer and Pharmaceutical Sciences, King's College London, London SE1 9NH, UK
3. Programa de Pós-graduação, em Biotecnologia Aplicada à Saúde da Criança e do Adolescente; Instituto de Pesquisa Pelé Pequeno Príncipe, Curitiba 80230-020, PR, Brazil; elizabeth.fernandes@pelepequenoprincipe.org.br
4. School of Bioscience Education, Faculty of Life Sciences & Medicine, King's College London, London SE1 1UL, UK

* Correspondence: ian.mcfadzean@kcl.ac.uk (I.M.); sue.brain@kcl.ac.uk (S.D.B.); Tel: +44-207-848-6053 (I.M.); +44-207-848-4453 (S.D.B.)

Gene	Primer sequence	Amplicon size (bp)	Accession number
Acta1	F: 5'-CTAAATCCAAGTCCTGCAAGTG-3'<br>R: 5'-ACATGGTGTCTAGTTTCAGAGG-3'	80	NM_001272041.1
Actb	F: 5'-CACAGCTTCTTTGCAGCTCCTT-3'<br>R: 5'-TCAGGATACCTCTCTTGCTCT-3'	250	NM_007393.3
B2m	F: 5'-GTCGCTTCAGTCGTCAGCA-3'<br>R: 5'-TTGAGGGGTTTTCTGGATAGCA-3'	96	NM_009735.3
Col1a1	F: 5'-TCTGACTGGAAGAGCGGAGAG-3'<br>R: 5'-AGACGGCTGAGTAGGGAACA-3'	120	NM_007742.4
Col1a2	F: 5'-TGGATACGCGGACTCTGTTG-3'<br>R: 5'-CCCTTTCGTACTGATCCCGATT-3'	85	NM_007743.3
Hprt	F: 5'-CCTGGTTCATCATCGCTAATC-3'<br>R: 5'-TCCTCCTCAGACCGCTTTT-3'	90	NM_013556.2
Mhy7	F: 5'-CACCTACCAGACAGAGGAAGA-3'<br>R: 5'-GGAGCTGGGTAGCACAAGA-3'	279	NM_080728.2
Nppa	F: 5'-GGATTTCAAGAACCTGCTAGACC-3'<br>R: 5'-GCAGAGCCCTCAGTTTGCT-3'	242	NM_008725.3
Tgfb1	F: 5'-TCAGACATTCGGGAAGCAGT-3'<br>R: 5'-GCCCTGTATTCCGTCTCCTTG-3'	237	NM_011577.2
Trpc1	F: 5'-TTGCGTAGATGTGCTTGGGA-3'<br>R: 5'-CTGCGGACTGACAACCGTA-3'	101	NM_011643.2
Trpc3	F: 5'-TTCAACGCCTCGGACAGATT-3'<br>R: 5'-AGACCACATCATCCCAAGAACC-3'	150	NM_019510.2
Trpc4	F: 5'-TACGGAAACCCCATCGGAAC-3'<br>R: 5'-AGTCCATCATCATCTCCGCAA-3'	128	NM_016984.3<br>NM_001253682.1<br>NM_001253683.1
Trpc5	F: 5'-AGATAAAGGAAATGTGGGATGGTG-3'<br>R: 5'-AGGAAATAGTTGCCAGGTAGAGG-3'	102	NM_009428.3
Trpc6	F: 5'-CCAGCTTCCGGGGTAATGAA-3'<br>R: 5'-TAGCATCTTCCGCACCACTG-3'	191	NM_013838.2<br>NM_001282087.1<br>NM_001282086.1
Trpc7	F: 5'-CTCCTTCACTGCGAGGTTCA-3'<br>R: 5'-ACCACTTATCCCTGGCGTAG-3'	143	NM_012035.3<br>NM_001302372.1<br>NM_001302373.1

Supplemental Table S1. List of genes and their respective primers for quantitative PCR. Forward (F) and reverse (R) primers were purchased from Sigma-Aldrich, UK. Where applicable, primers were generated to target all known splice variants of that gene. *Acta1*, skeletal α-actin; *Actb*, β-actin; *B2m*, β-2-microglobulin; *Col1a1*, collagen type I α1; *Col1a2*, collagen type I α2; *Hprt*, hypoxanthine guanine phosphoribosyl transferase; *Mhy7*, β-myosin heavy chain; *Nppa*, atrial natriuretic peptide; *Tgfb1*, transforming growth factor-β1; *Trpc*, transient receptor potential canonical.

	TRPC5 WT	TRPC5 KO
Heart weight (mg)	128.18 ± 5.83	134.66 ± 5.35
HW (mg) / TL (mm)	7.16 ± 0.30	7.48 ± 0.27
LV mass	115.26 ± 7.01	120.41 ± 8.81
LV (mg)/ TL (mm)	6.55 ± 0.41	6.76 ± 0.44
LVPWd (mm)	0.91 ± 0.05	0.85 ± 0.03
LVPWs (mm)	1.17 ± 0.05	1.14 ± 0.04
IVSd (mm)	1.11 ± 0.08	1.07 ± 0.07
IVSs (mm)	1.53 ± 0.06	1.46 ± 0.09
LVAWd (mm)	1.03 ± 0.08	1.10 ± 0.07
LVAWs (mm)	1.43 ± 0.10	1.53 ± 0.13
LVIDd (mm)	3.69 ± 0.13	3.98 ± 0.06*
LVIDs (mm)	2.46 ± 0.17	2.67 ± 0.10
LV volume d (µl)	58.39 ± 4.47	69.52 ± 2.35*
LV volume s (µl)	22.69 ± 3.33	26.87 ± 2.33

Supplemental Table S2. Cardiac mass and dimension characterisation in naïve WT and TRPC5 KO mice. Heart weight (HW) and left ventricle (LV) mass were normalised to tibia length (TL). LV posterior wall (LVPW), intraventricular septum (IVS), LV anterior wall (LVAW), LV internal dimension (LVID) thicknesses, and LV volumes were assessed via 2-dimensional echocardiography (n=8-14). Measurements obtained during diastole (d) and systole (s). Data shown as mean ± s.e.m. *$p<0.05$ as determined by unpaired Students *t*-test.

	Sham		*AAB*	
	**TRPC5 WT**	**TRPC5 KO**	**TRPC5 WT**	**TRPC5 KO**
**4 week**				
LVAWd (mm)	1.14 ± 0.05	1.10 ± 0.07	1.25 ± 0.10	1.31 ± 0.07
LVAWs (mm)	1.39 ± 0.07	1.55 ± 0.07	1.73 ± 0.07*	1.78 ± 0.07
IVSd (mm)	1.08 ± 0.11	1.14 ± 0.05	1.21 ± 0.10	1.41 ± 0.06
IVSs (mm)	1.47 ± 0.16	1.47 ± 0.05	1.54 ± 0.11	1.87 ± 0.08*,#
LVIDd (mm)	3.74 ± 0.08	4.04 ± 0.08	3.98 ± 0.11	4.16 ± 0.14
LVIDs (mm)	2.67 ± 0.13	2.85 ± 0.08	2.83 ± 0.13	2.82 ± 0.15
LV volume d (µl)	59.84 ± 3.07	72.16 ± 3.56	69.84 ± 4.56	77.65 ± 5.78
LV volume s (µl)	26.67 ± 3.16	31.08 ± 2.16	31.21 ± 3.65	31.14 ± 3.99
Stroke volume (µl)	33.91 ± 2.20	41.08 ± 2.77	38.63 ± 2.04	46.51 ± 2.13
Cardiac output (ml/min)	16.24 ± 2.05	17.60 ± .08	17.46 ± 1.07	22.15 ± 2.13
**10 week**				
LVAWd (mm)	1.09 ± 0.09	1.16 ± 0.05	1.26 ± 0.07	1.26 ± 0.12
LVAWs (mm)	1.57 ± 0.09	1.48 ± 0.06	1.73 ± 0.09	1.85 ± 0.09
IVSd (mm)	1.10 ± 0.08	1.16 ± 0.06	1.25 ± 0.08	1.36 ± 0.07
IVSs (mm)	1.42 ± 0.09	1.54 ± 0.09	1.64 ± .0.09	1.84 ± 0.08
LVIDd (mm)	3.90 ± 0.06	4.17 ± 0.10	4.02 ± 0.10	4.07 ± 0.10
LVIDs (mm)	2.76 ± 0.06	2.91 ± 0.16	2.78 ± 0.13	2.74 ± 0.11
LV volume d (µl)	66.08 ± 2.48	77.88 ± 4.83	71.59 ± 4.48	73.62 ± 4.08
LV volume s (µl)	28.63 ± 1.59	33.98 ± 4.75	30.24 ± 3.86	28.47 ± 2.81
Stroke volume (µl)	38.09 ± 2.51	43.90 ± 1.85	41.35 ± 1.43	45.16 ± 2.61
Cardiac output (ml/min)	16.04 ± 1.27	21.34 ± 1.25	18.45 ± 1.10	20.63 ± 1.65

Supplemental Table S3. Left ventricle dimensions in mice subjected to pressure overload. Cardiac hypertrophy was developed in mice for 4 weeks (n=6-9) and 10 weeks (n=10-12) following abdominal aortic banding (AAB). Left ventricle anterior wall (LVAW), intraventricular septum (IVS), LV internal dimension (LVID) thicknesses, and LV volumes were assessed via 2-dimensional echocardiography. Measurements obtained during diastole (d) and systole (s). Data shown as mean ± s.e.m. *$p<0.05$ vs respective sham, #$p<0.05$ vs WT AAB as determined by two-ANOVA followed by the *Bonferroni post-hoc* correction.

	Sham		*AAB*	
	**TRPC5 WT**	**TRPC5 KO**	**TRPC5 WT**	**TRPC5 KO**
**4 week**				
Trpc1	45.22 ± 16.38	48.47 ± 8.98	50.40 ± 4.85	47.46 ± 7.20
Trpc3	93.12 ± 21.07	80.59 ± 10.94	96.36 ± 18.63	84.96 ± 9.36
Trpc4	20.05 ± 10.33	19.30 ± 5.03	16.17 ± 3.89	17.09 ± 4.04
Trpc6	4.33 ± 1.52	3.51 ± 0.54	6.10 ± 0.68	4.37 ± 1.05
Trpc7	44.47 ± 17.39	43.54 ± 5.92	47.17 ± 2.47	43.63 ± 11.49
**10 week**				
Trpc1	58.83 ± 2.53	56.19 ± 4.71	49.75 ± 2.19	58.53 ± 3.10
Trpc3	99.99 ± 5.42	99.05 ± 9.11	101.90 ± 5.58	87.08 ± 6.73
Trpc4	26.23 ± 2.80	25.78 ± 3.04	24.76 ± 2.39	22.90 ± 2.59
Trpc6	4.63 ± 0.45	3.38 ± 0.25	4.58 ± 0.36	5.04 ± 0.41
Trpc7	74.80 ± 6.28	76.52 ± 4.69	74.78 ± 4.54	74.06 ± 5.98

Supplemental Table S4. mRNA expression profile of canonical subunits in left ventricles of mice subjected to pressure overload. *Trpc1*, *Trpc3*, *Trpc4*, *Trpc6* and *Trpc7* gene expression were assessed via RT-qPCR in cardiac left ventricles from mice subjected to 4 weeks (n=5-7) and 10 weeks (n=10-12) of abdominal aortic banding (AAB). Data was normalised to the reference genes *B2m* (β-2-microglobulin) and *Hprt* (hypoxanthine guanine phosphoribosyl transferase) and expressed as copies/μl. Data shown as mean ± s.e.m. and analysed using two-ANOVA followed by the *Bonferroni post-hoc* correction.

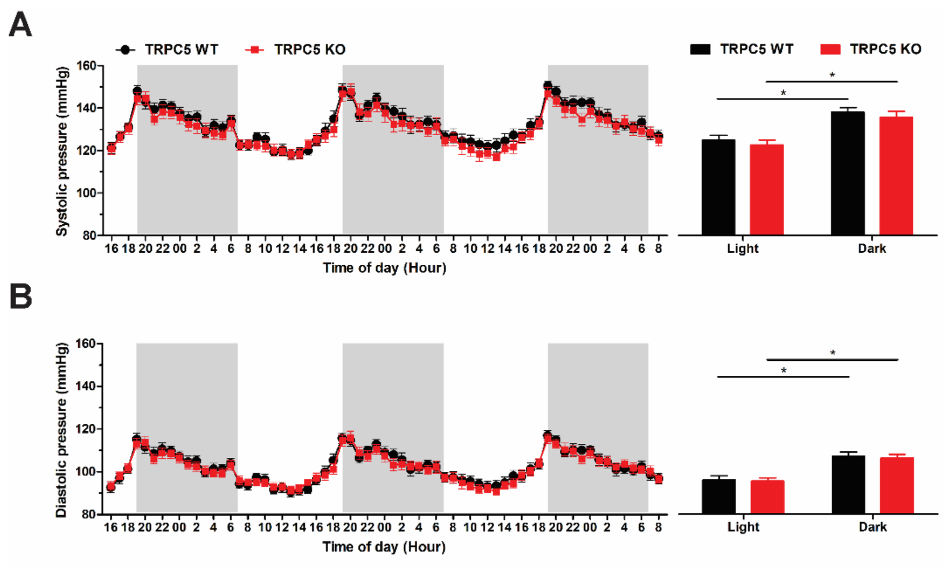

Supplemental Figure S1. **Blood pressure parameters in naïve WT and TRPC5 KO mice.** (A) Systolic and (B) diastolic blood pressure recordings were obtained from radio-telemetered mice over a 2 light/3 dark period. Shaded regions depict dark phase recordings and bar charts illustrate average for the light and dark periods (n=19-20). Data shown as mean ± s.e.m. *p<0.05 as determined by two-ANOVA followed by the *Bonferroni post-hoc* correction.

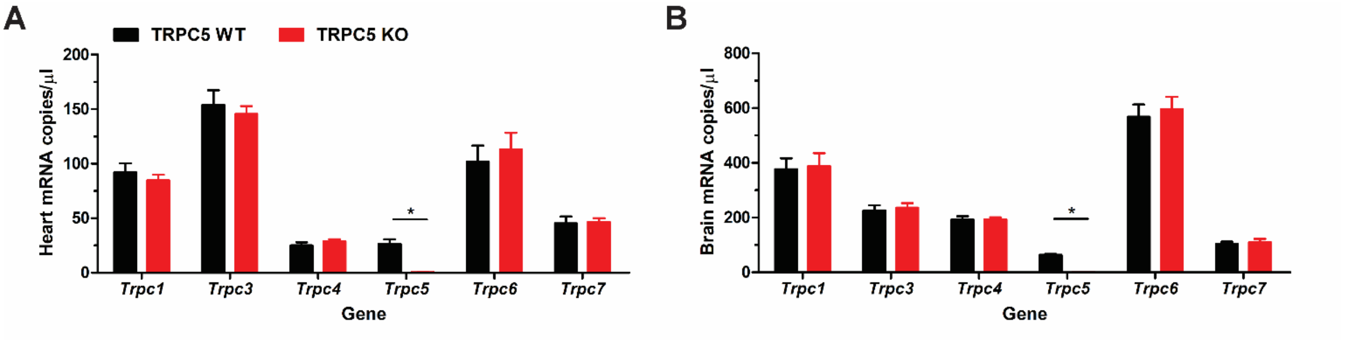

Supplemental Figure S2. mRNA expression profile of the TRPCs in tissue collected from naïve WT and TRPC5 KO mice. mRNA was extracted from (A) whole hearts and (B) whole brains of mice. Data was normalised to the reference genes *Actb* (β-actin), *B2m* (β-2-microglobulin) and *Hprt* (hypoxanthine guanine phosphoribosyl transferase) and expressed as copies/µl. Data shown as mean ± s.e.m. *p<0.05 as determined by unpaired Students *t*-test. n=8 for all groups.

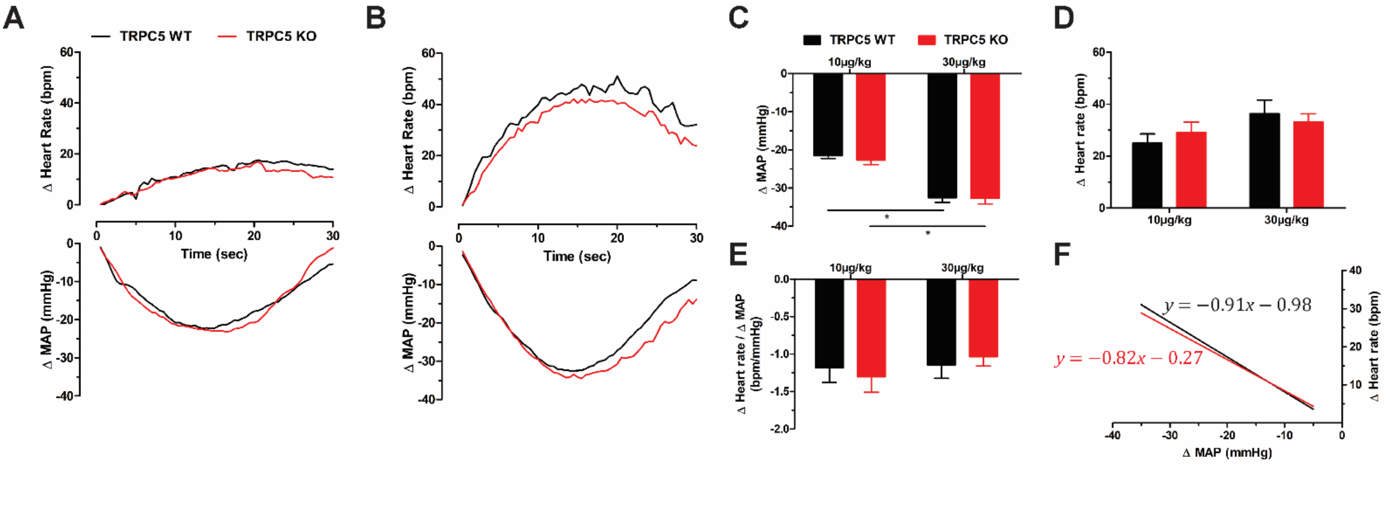

Supplemental Figure S3. Reflex response to SNP administration in TRPC5 KO mice. Changes in mean arterial pressure and heart rate were evaluated in mice following sodium nitroprusside (SNP) *i.v.* (n=7). Representative traces depicting changes in mean arterial pressure (MAP) and heart rate following (A) 10µg/kg and (B) 30µg/kg SNP. Peak changes in (C) MAP, (D) heart rate were assessed, and (E) ratio of heart rate over MAP was used a measure of overall baroreflex sensitivity. (F) Linear regression for the tachycardiac response following SNP. Data shown as mean ± s.e.m. *p<0.05 as determined by two-ANOVA followed by the *Bonferroni post-hoc* correction.

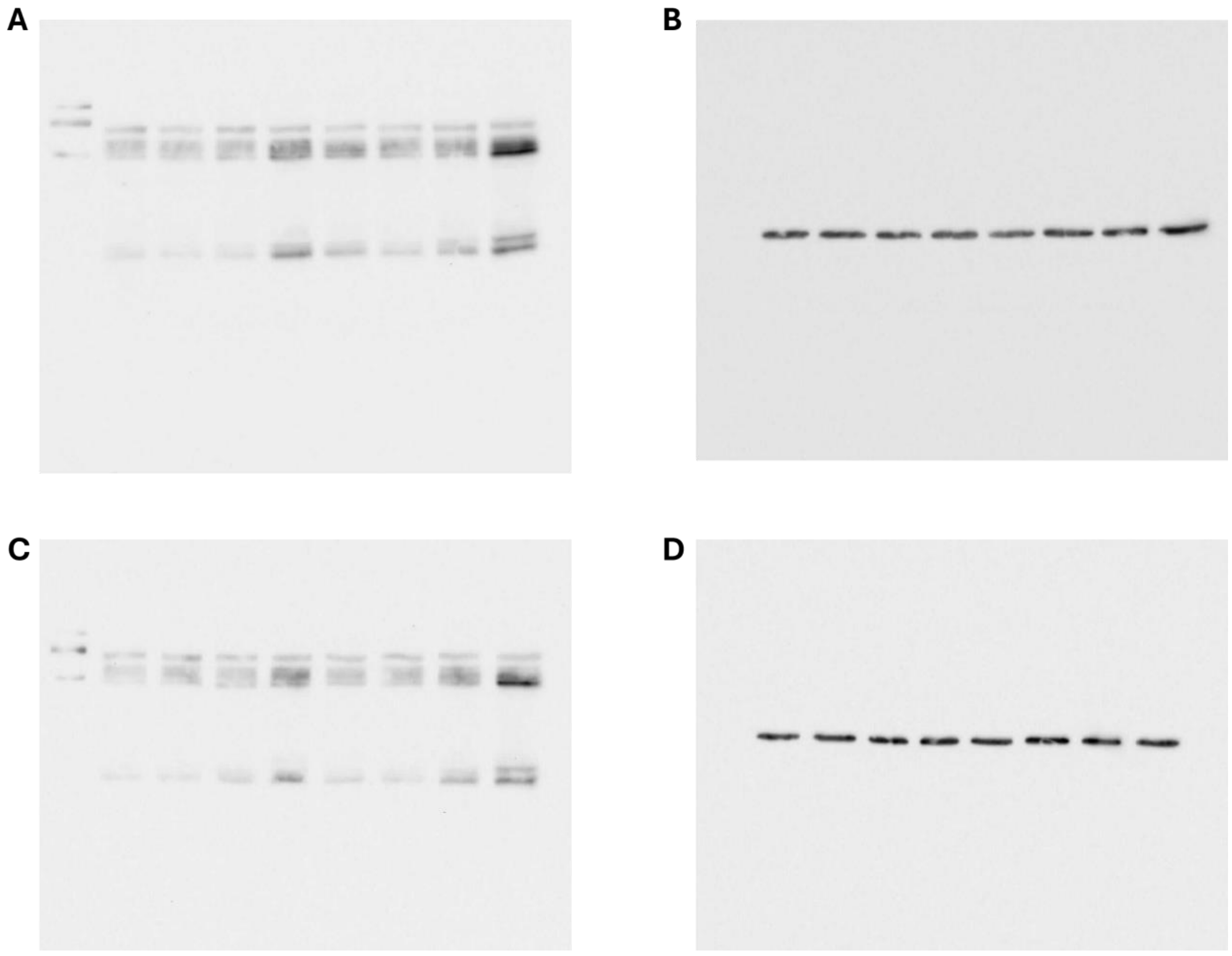

Supplemental Figure S4. gp91(phox) original Western blot. Original Western blots labelled for (A) gp91(phox) and (B) GAPDH protein expression from cardiac left ventricles of WT and TRPC5 KO mice subjected to 4 weeks of abdominal aortic banding (AAB) or sham. Original Western Blots labelled for (C) gp91(phox) and (D) GAPDH protein expression from cardiac left ventricles of WT and TRPC5 KO mice subjected to 10 weeks of abdominal aortic banding (AAB) or sham.

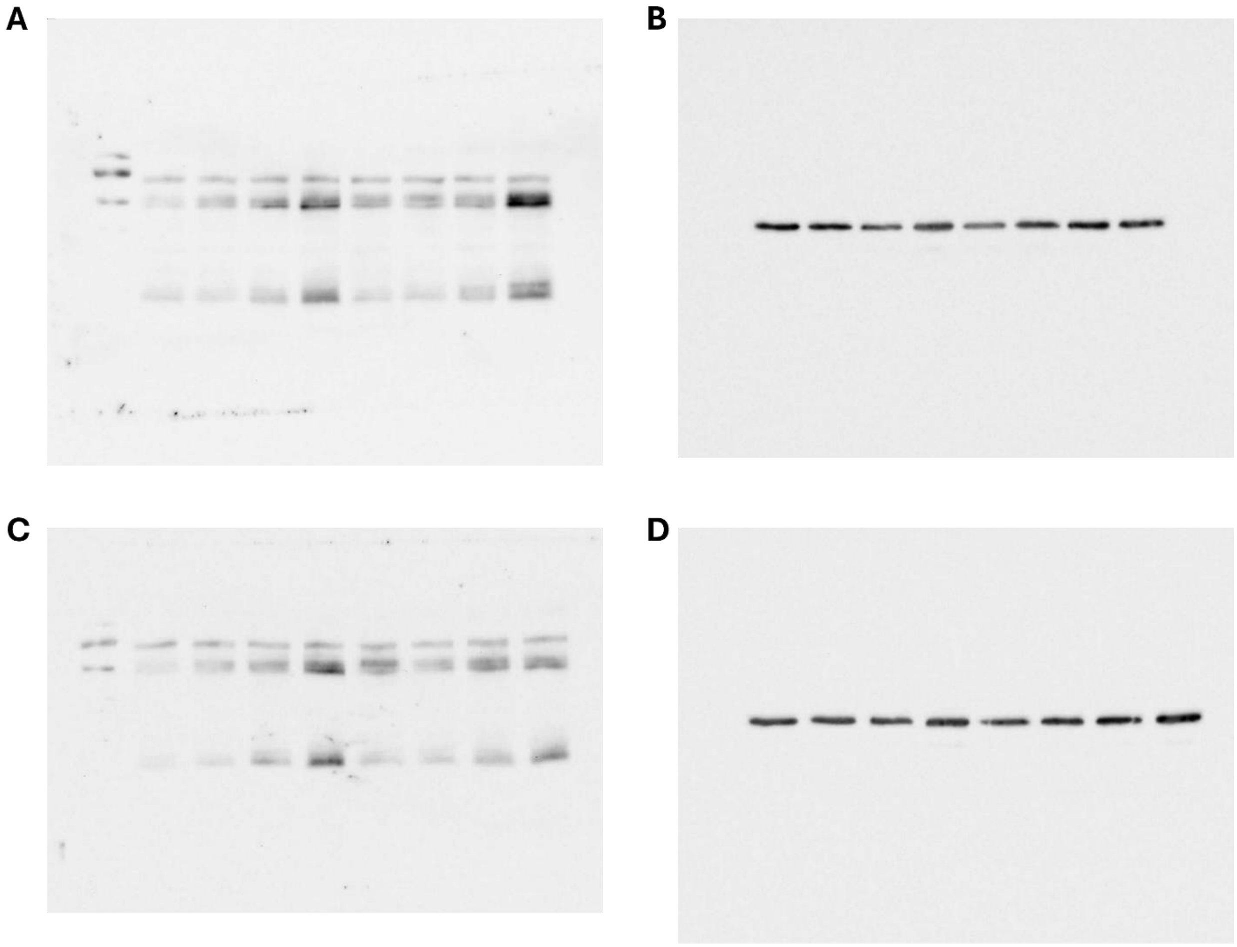

Supplemental Figure S5. Nitrotyrosine original Western blot. Original Western Blots labelled for (A) nitrotyrosine and (B) GAPDH protein expression from cardiac left ventricles of WT and TRPC5 KO mice subjected to 4 weeks of abdominal aortic banding (AAB) or sham. Original Western Blots labelled for (C) nitrotyrosine and (D) GAPDH protein expression from cardiac left ventricles of WT and TRPC5 KO mice subjected to 10 weeks of abdominal aortic banding (AAB) or sham.

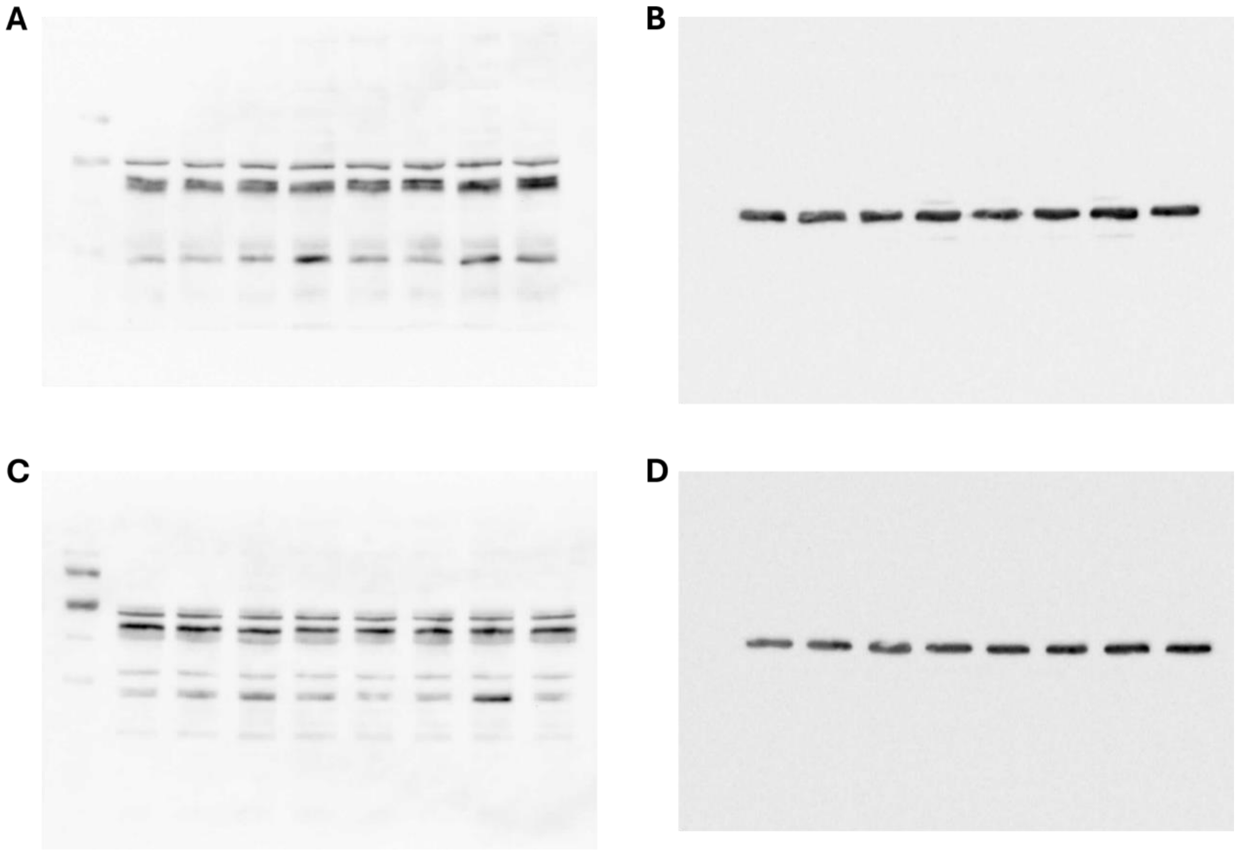

Supplemental Figure S6. Heme oxygenase 1 (HO-1) original Western blot. Original Western Blots labelled for (A) HO-1 and (B) GAPDH protein expression from cardiac left ventricles of WT and TRPC5 KO mice subjected to 4 weeks of abdominal aortic banding (AAB) or sham. Original Western Blots labelled for (C) HO-1 and (D) GAPDH protein expression from cardiac left ventricles of WT and TRPC5 KO mice subjected to 10 weeks of abdominal aortic banding (AAB) or sham.
